# Genome sequence analysis of new plum pox virus isolates from Japan

**DOI:** 10.1186/s13104-021-05683-9

**Published:** 2021-07-10

**Authors:** Tomoaki Mori, Chiaki Warner, Serika Ohno, Koichi Mori, Takamasa Tobimatsu, Takashi Sera

**Affiliations:** Department of Applied Chemistry and Biotechnology, Graduate School of Interdisciplinary Science and Engineering in Health Systems, Okayama University, Tsushima-Naka, Kita-ku, Okayama, 700-8530 Japan

**Keywords:** Plum pox virus, Complete genome sequence, Phylogenetic analysis, Sequence alignment analysis, Genetic variation

## Abstract

**Objective:**

To find mutations that may have recently occurred in *Plum pox virus* (PPV), we collected six PPV-infected plum/peach trees from the western part of Japan and one from the eastern part. After sequencing the full-length PPV genomic RNAs, we compared the amino acid sequences with representative isolates of each PPV strain.

**Results:**

All new isolates were found to belong to the PPV-D strain: the six isolates collected from western Japan were identified as the West-Japan strain while the one collected from eastern Japan as the East-Japan strain. Amino acid sequence analysis of these seven isolates suggested that the 1407th and 1529th amino acid residues are characteristic of the West-Japan and the East-Japan strains, respectively. Comparing them with the corresponding amino acid residues of the 47 non-Japanese PPV-D isolates revealed that these amino acid residues are undoubtedly unique. A further examination of the relevant amino acid residues of the other 210 PPV-D isolates collected in Japan generated a new hypothesis regarding the invasion route from overseas and the subsequent diffusion route within Japan: a PPV-D strain might have invaded the western part of Japan from overseas and spread throughout Japan.

**Supplementary Information:**

The online version contains supplementary material available at 10.1186/s13104-021-05683-9.

## Introduction

*Plum pox virus* (PPV), an RNA virus belongs to the genus *Potyvirus* in the family *Potyviridae*, causes a serious viral disease known as a pox of plum or Sharka in stone fruit trees (plums, apricots, peaches, nectarines, almonds, sweet cherries, tart cherries, and so on) and was first reported in plum trees in Bulgaria in 1915 [1]. PPV infects stone fruit trees such as plums, apricots, and peaches via aphid and graft inoculation [2]. As the infection progresses, the fruits may be distorted or depressed on the surface, and the premature fruits may drop [3]. The viral disease is a serious problem for the stone fruit industry: the combined cost of crop damage caused by the infection has been estimated to exceed $10 billion over the last 30-years worldwide [4].

PPV is widespread and distributed throughout European countries, the Mediterranean coast, the Middle East, South America, North America, and Asia [5]. In Japan, the first PPV was discovered in 2009 in Ome, Tokyo (eastern Japan). At this time, the full genome sequence was determined and identified as a PPV-D strain [6]. In response to the infection, the Japanese government conducted a nationwide survey. The survey revealed that PPV had spread not only in eastern Japan but also to western Japan, including the Itami City, Hyogo prefecture. In subsequent years, annual surveys found 173 types of PPV belonging to the PPV-D strain isolated from the leaves of infected stone fruit trees between 2009 and 2014 in Japan. The complete genome sequences of the 173 PPV strains found, except for the terminal 24–26 nt, have been reported [7]. To find mutations that may have occurred in PPV after 2014, we collected PPV-infected trees in Japan between 2017 and 2018, isolated PPVs and analyzed these complete genome sequences.

## Main text

### Methods

#### PPV-infected trees

Six PPV-infected plum/peach potted small trees from the western part of Japan and one from the eastern part were obtained with permission from the Ministry of Agriculture, Forestry and Fisheries (MAFF) of Japan. Viral infection was confirmed by MAFF Plant Protection Stations with both immunochromatography and RT-LAMP assays.

#### Analysis of PPV genome sequence

Three partially overlapping DNA fragments (3587, 3856, and 3583 bp) were amplified from each PPV genome by RT-PCR and sequenced as previously described [6]. The total RNA was extracted from PPV-infected leaves using RNeasy Plant Mini Kit (QIAGEN, Valencia, CA) and digested with DNase I (ThermoFisher SCIENTIFIC, Waltham, MA). Reverse transcription reactions were performed using SuperScript III Reverse Transcriptase (ThermoFisher SCIENTIFIC) and three primer sets (PPV3587R, PPV6683R, and PPV9786R). The resulting three products were amplified with thermocycler steps consisting of 95 °C for 2 min, 30 cycles of 95 °C for 20 s, 55 °C for 20 s and 72 °C for 2 min, and a final extension of 72 °C for 3 min using PfuUltraII Fusion HS DNA Polymerase (Agilent Technologies, Santa Clara, CA) and the primer sets of PPV1F and PPV3587R, PPV2828F and PPV6683R, and PPV6204F and PPV9786R, respectively. Each RT-PCR product was purified using the QIAquick Gel Extraction Kit (QIAGEN) and sequenced using 22 sequencing primers. The complete genome sequences were deposited in the DDBJ/GenBank/EMBL database and the Accession Numbers are listed in Section “Availability of data and materials”. All primers’ information was listed in Additional file 1: Table S1.

#### Alignment analysis of amino acid sequences of PPV polyproteins

The polyprotein amino acid sequences of the seven new PPV isolates obtained in this study were aligned with those of each representative isolate of twelve distinct PPV strains [D (East-Japan), D (West-Japan), D (Canada), An, Rec, M, T, EA, W, C, CR, and CV] (Additional file 2: Table S2) or 47 non-Japanese PPV-D isolates (Additional file 3: Table S3) available from the NCBI database and analyzed using Clustal W.

#### Phylogenetic analysis

Genomic nucleotide sequences of PPV isolates used in this study were aligned with Clustal W or MUSCLE within the MEGA X software [8] (see Section “Availability of data and materials” for the accession numbers). Phylogenetic trees were generated in MEGA X using the Maximum-likelihood method under the GTR + G + I model. Bootstrap values were obtained from 1000 iterations.

### Results and discussion

We collected six infected trees in western Japan and one infected tree in eastern Japan between 2017 and 2018 to find mutations that may have occurred in PPV after 2014 (see Additional file 4: Figure S1 for these collection locations). Using a method from Namba’s group [6], we isolated PPVs (designated PPV1, PPV3, PPV4, PPV5, PPV6, PPV11, and PPV12) from each infected leaf and determined their complete nucleotide sequences (see Additional file 5: Table S4 for details) of the reverse-transcribed genomes except 24 nt at the 5′ end and 26 nt at the 3′ end, as reported previously reported [6] (see “Methods” Section for the detailed procedure). None of the isolates were found in the BLAST search database and they have been demonstrated to be new mutant strains that have not yet been reported.

To investigate which PPV strain the new isolates belong to, we compared them with the amino acid sequence of representative isolates (Additional file 2: Table S2; [7]) of each of the current 10 PPV strains; PPV-D (Dideron), PPV-M (Marcus), PPV-Rec (Recombinant), PPV-EA (El Amar), PPV-C (Cherry), PPV-W (Winona), PPV-T (Turkey), PPV-CR (Cherry Russian), PPV-An (Ancestor), and PPV-CV (Cherry Volga) with Ou1 (a representative isolate of the East-Japan population) and It2079 (that of the West-Japan population) [3, 7, 9]. As a result, it turned out that all our new isolates have the best homology (99% or more) with a PPV-D Canadian isolate (Vulcan) (Table 1). Further analysis of nucleotide sequence homology (Table 1) and phylogenetic tree mapping (Additional file 6: Figure S2) has revealed that all six isolates (PPV1, PPV3, PPV4, PPV5, PPV6, and PPV11) collected in western Japan, and the one isolate (PPV12) from eastern Japan belong to the West-Japan and East-Japan populations, respectively.

**Table 1:** Percent identity of amino acid (aa) sequences for polyproteins (total 3140 aa) and nucleotide (nt) sequences for the complete genome (total 9736 nt) of new isolates and other PPV strains

Amino acid identity (%)							
	PPV1	PPV3	PPV4	PPV5	PPV6	PPV11	PPV12
City isolated:	Kawanishi	Itami	Itami	Amagasaki	Itami	Kawanishi	Ome
Region in Japan:	West	West	West	West	West	West	East
PPV-D: Canada (Vulcan)	99.33	99.24	99.20	99.20	99.01	99.20	99.11
PPV-D: East-Japan (Ou1)	99.68	99.62	99.59	99.59	99.43	99.62	**99.62**
PPV-D: West-Japan (It2079)	**99.81**	**99.84**	**99.81**	**99.84**	**99.71**	**99.87**	**99.62**
PPV-Rec (BOR-3)	98.15	98.03	97.80	97.99	97.83	97.99	98.03
PPV-M (PS)	96.50	96.53	96.43	96.43	96.37	96.46	96.43
PPV-T (AbTk)	96.46	96.43	96.43	96.34	96.21	96.37	96.34
PPV-An (AL11pl)	95.06	95.06	95.00	95.03	94.90	94.97	94.94
PPV-EA (El Amar)	91.21	91.18	90.92	91.08	91.08	91.18	91.18
PPV-C (BY181)	88.60	88.66	88.28	88.47	88.44	88.60	88.63
PPV-CR (RU-30sc)	88.50	88.54	88.28	88.44	88.44	88.57	88.54
PPV-W (LV-145bt)	88.38	88.54	88.22	88.38	88.28	88.41	88.41
PPV-CV (Tat-2)	88.03	87.96	87.80	87.87	87.87	87.96	87.87

Nucleic acid identity (%)							
New isolate:	PPV12						
PPV-D: East-Japan (Ou1)	**99.52**						
PPV-D: West-Japan (It2079)	99.31						

Percentages in bold indicate the highest values

**Fig. 1:**
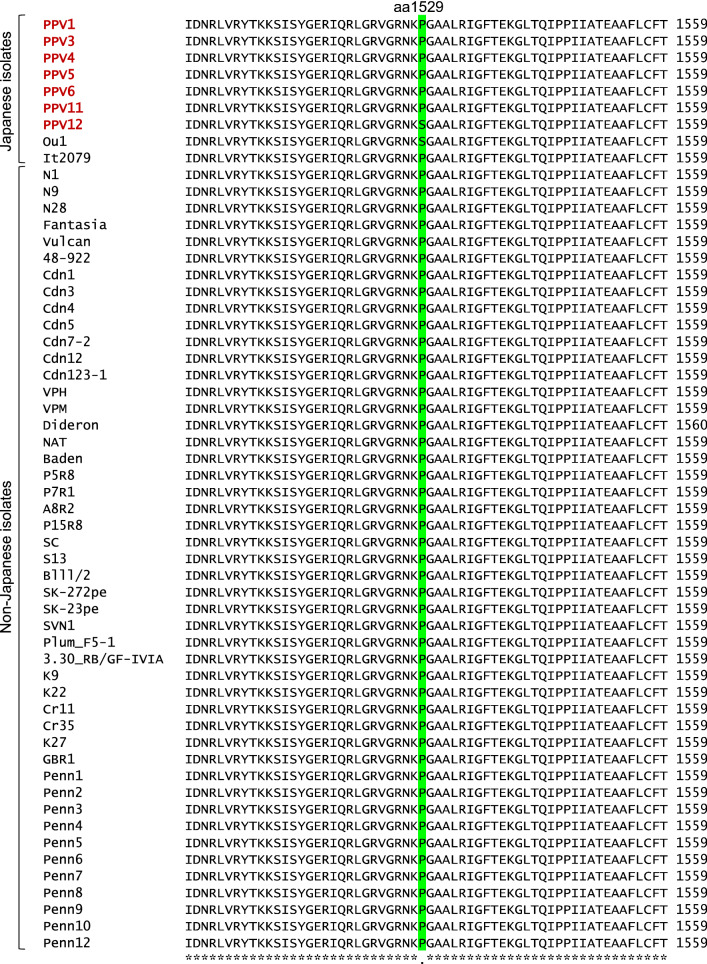
Multiple amino acid sequence alignment of Japanese PPV-D isolates and 47 non-Japanese PPV-D isolates around the 1529th amino acid residues. The amino acid residues at 1529 are highlighted with green. Symbols denote the degree of conservation observed in each column: “*” (identical residues in all sequence), “.” (weakley conserved column)

Next, we characterized our new isolates by comparing them with 47 non-Japanese PPV-D isolates (Additional file 3: Table S3) whose complete genomic sequences have been determined. A phylogenetic tree analysis of the nucleotide sequences revealed that our new isolates formed distinct clades with Ou1 or It2079 and were genetically close to Western European PPV-D isolates (especially to the UK isolate BGR1) (Additional file 7: Figure S3). Further, we performed an alignment analysis of these amino acid sequences. As a result, we newly identified two strain characteristic amino acid residues in addition to the 2635th amino acid that is peculiar to the West-Japan population, previously reported by Namba’s group [7]. The first is the 1407th amino acid (Additional file 8: Figure S4). This amino acid residue was Gly in all 47 non-Japanese isolates, the West-Japan isolates It2079 and PPV1 (our new isolate), and the East-Japan isolates Ou1 and PPV12 (our new isolate). In our PPV3, PPV4, PPV5, PPV6, and PPV11 isolates, the 1407th amino acid was Ser, suggesting, for the first time, that this amino acid residue is a new characteristic of the West-Japan population. The second is the 1529th amino acid (Fig. 1). This amino acid residue was Ser only in the East-Japan isolates Ou1 and PPV12, and was Pro in all other isolated strains including non-Japanese PPV-D isolates. In Japan, PPV was first discovered as a PPV-D strain in the suburbs of Tokyo, so invasion from overseas (Europe: [6, 7]) into the suburbs of Tokyo has been considered to be the most likely transmission route [7]. Interestingly, this amino acid residue of the West-Japan population, but not the East-Japan population, was identical to that of the non-Japanese PPV-D isolates.

To further confirm the characteristics of these newly discovered amino acid residues (1407 and 1529), we performed amino acid sequence alignment analysis with 134 East-Japan and 76 West-Japan isolates (Additional file 9: Table S5) that had already been reported [7]. From that analysis, new findings were obtained for each amino acid residue. First, the amino acid residue at position 1407 was Gly (GGT) in all the East-Japan isolates containing PPV12 and 46 West-Japan isolates, including PPV1. On the other hand, in 30 West-Japan isolates including PPV3, PPV4, PPV5, PPV6, and PPV11, the amino acid residue at this position was Ser (AGT: mutated nucleotide underlined). Further mutations were observed in the West-Japan population: in six West-Japan isolates, it was changed to Asn (AAT: mutated nucleotide underlined). Thus, the 1407th amino acid residue variation was a phenomenon only observed in the West-Japan population. In addition, an interesting finding was obtained at amino acid residue 1529. This amino acid residue was Pro (CCA) in all West-Japan isolates including PPV1, PPV3, PPV4, PPV5, PPV6, and PPV11. On the other hand, most of the East-Japan isolates (131 isolates among 135 ones), including PPV12, were Ser (TCA). As mentioned above, all non-Japanese PPV-D strains that are thought to have invaded Japan were Pro (CCA). If the East-Japan type (where the 1529th amino acid residue is Ser, but not Pro) had spread throughout Japan, this variation of the amino acid residue could not be explained. The variation that we found in this study suggests that a non-Japanese PPV-D strain might have first invaded western Japan centering around Osaka and Kobe (Additional file 4: Figure S1), and then spread further. It could thus be considered that after the PPV-D strain subsequently moved to eastern Japan, due to the mutation from CCA to TCA during its diffusion in eastern Japan, the PPV-D strain having Ser at position 1529 was selected for some reason and then spread throughout eastern Japan. As evidence, in the same area where the East-Japan type Ak1 having Pro at position 1529 were found, the strains (Ak2, Ak3, Ak4) having Ser at position 1529 have also been isolated (Additional file 4: Figure S1). Alternatively, a non-Japanese PPV-D strain with Ser at position 1529 that have not yet been isolated may have independently invaded eastern Japan.

Amino acid residues 1407 and 1529 are present in the CI protein. We searched the literature for the effects of these two residues on the structure/function of CI proteins and their functions such as the infectivity of PPV, but we could not find any reports. The role of these two amino acid residues in the structure and function of PPV remains to be elucidated.

**Fig. 2:**
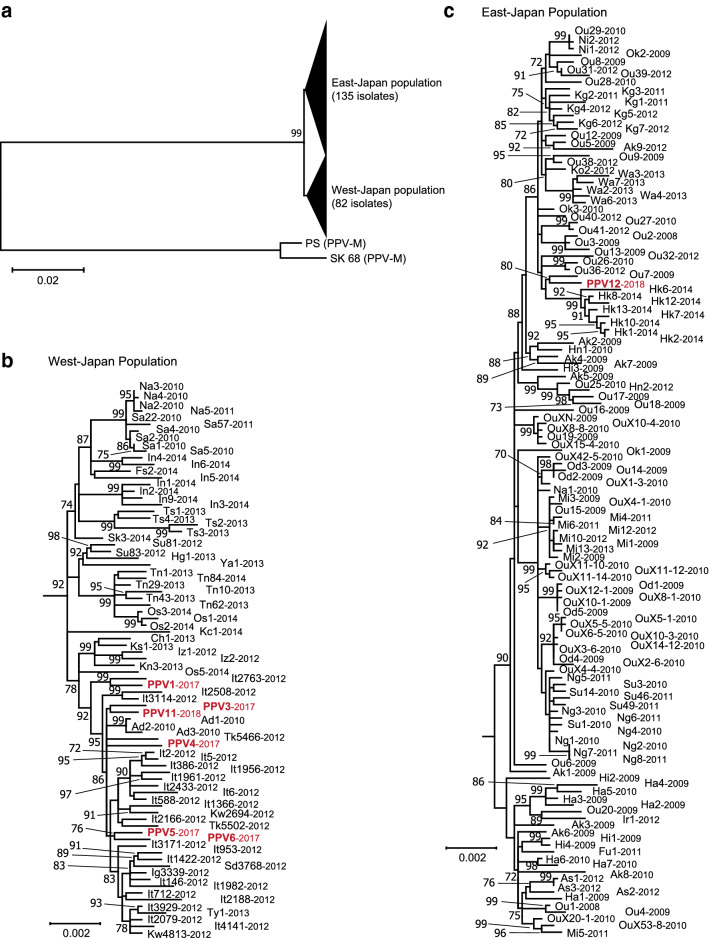
Phylogenetic tree generated by the maximum-likelihood method with 1000 bootstrap replicates based on complete genome sequences of the seven new isolates (shown as red letters) and 210 previously reported Japanese PPV-D isolates (134 isolates of East-Japan population and 76 isolates of West-Japan population) [7]. Two PPV-M isolates (PS: AJ243957 and SK 68: M92280) were used as an outgroup. Branch lengths indicate the number of nucleotide differences per site, and numbers at nodes indicate bootstrap values greater than 70. For the sake of clarity, interior branches representing distinct clusters are collapsed into filled triangles (**a**). The detailed topology of these clusters for the West- and East-Japan populations are shown in **b** and **c**, respectively. The number after each isolate name in **b** and **c** represents the year of collection

Finally, a phylogenetic tree was created using the seven isolates collected in this study along with the 210 previously reported Japanese isolates and the two PPV-M isolates (PS and SK68) as an outgroup (Fig. 2). As a result, the PPV12 isolate formed the same clade as the previously reported East-Japan type isolate Ou7. In contrast, PPV3 and PPV11, PPV5 and PPV6, and PPV4 formed distinct clades, respectively. This suggests that PPV strains may have mutated between 2014 (the last year of the national survey) and 2017–2018 (time of collection for the new isolates.

In summary, in this study, we have determined the whole nucleotide sequences of seven new isolates of PPV-D strains recently collected in Japan, indicating that PPV has been continuously mutating in Japan. Also, by comparing these new isolates with the non-Japanese PPV-D isolates, we have identified two new residues, the 1407th and 1529th amino acid residues, as characteristic residues of Japanese PPV-D strains in addition to the 2635th amino acid residue already reported [7]. The analysis strongly suggests that the areas of invasion in Japan from overseas include the area near Tokyo in eastern Japan where PPV was first discovered and western Japan centering around Osaka and Kobe. Additional research is needed to further understand the route of PPV invasion into Japan and the route of diffusion within Japan.

### Limitations

We have identified two new residues, the 1407th and 1529th amino acid residues, as characteristic residues of Japanese PPV-D strains in addition to the 2635th amino acid residue already reported. However, we could not find any literature for the effects of these two residues on the structure/function of CI proteins and their functions such as the infectivity of PPV. Therefore, the role of these two amino acid residues in the structure and function of PPV will need to be elucidated.

## Supplementary Information

**Additional file 1: Table S1.** List of primers used in this study.

**Additional file 2: Table S2.** List of PPV strains used in this study.

**Additional file 3: Table S3.** List of non-Japanese PPV-D isolates used in this study.

**Additional file 4: Figure S1.** Map of the collection locations of new isolates and previously reported Japanese PPV-D isolates used for analysis or discussion in this study.

**Additional file 5: Table S4.** List of PPV strains isolated in this study.

**Additional file 6: Figure S2.** Phylogenetic tree generated by the maximum-likelihood method with 1000 bootstrap replicates based on complete genome sequences of new seven isolates and each representative isolate of PPV strains.

**Additional file 7: Figure S3.** Phylogenetic tree generated by the maximum-likelihood method with 1000 bootstrap replicates based on complete genome sequences of new seven isolates and 47 non-Japanese PPV-D isolates.

**Additional file 8: Figure S4.** Multiple amino acid sequence alignment of Japanese PPV-D isolates and 47 non-Japanese PPV-D isolates around the 1407th amino acid residues.

**Additional file 9: Table S5.** List of previously reported Japanese PPV-D isolates used in this study.

## Data Availability

The complete genome sequences of PPV1, PPV3, PPV4, PPV5, PPV6, PPV11, and PPV12 have been deposited in the DDBJ/GenBank/EMBL database and are available under the Accession Numbers from LC600459 to LC600465 (see Additional file 5: Table S4 for details). The names and Accession Numbers of each PPV strain’s isolate(s) used in this study are as follows: PPV-D (Ou1, AB545926; It2079, LC374999; Vulcan, AY912057), PPV-An (AL11pl, HF674399), PPV-Rec (BOR-3, AY028309), PPV-M (PS, AJ243957), PPV-T (AbTk, EU734794), PPV-EA (El Amar, DQ431465), PPV-W (LV-145bt, HQ670748), PPV-C (BY181, HQ840518), PPV-CR (RU-30sc, KC020126), and PPV-CV (Tat-2, MF447179) (see the Additional file 2: Table S2). The names and Accession Numbers of non-Japanese PPV-D isolates used in this study are as follows: N1, LC375128; N9, LC375129; N28, LC375130; Fantasia, AY912056; Vulcan, AY912057; 48-922, AY912058; Cdn1, AY953261; Cdn3, AY953262; Cdn4, AY953263; Cdn5, AY953264; Cdn7-2, AY953265; Cdn12, AY953266; Cdn123-1, AY953267; VPH, KP998124; VPM, KU948432; Dideron, X16415; NAT, D13751; Baden, KU508427; P5R8, LT600779; P7R1, LT600780; A8R2, LT600781; P15R8, LT600782; SC, X81083; S13, LC375131; BIII/2, GU461890; SK-272pe, HF585098; SK-23pe, LT158756; SVN1, LC375132; Plum_F5-1, LC331298; 3.30 RB/GF-IVIA, KJ849228; K9, KR006729; K22, KR006730; Cr11, KR028385; Cr35, KR028386; K27, KR028387; GBR1, LC375127; Penn1, AF401295; Penn2, AF401296; Penn3, DQ465242; Penn4, DQ465243; Penn5, EF640933; Penn6, EF640934; Penn7, EF640935; Penn8, EF640936; Penn9, EF640937; Penn10, EF640938; Penn12, EF640939 (see the Additional file 3: Table S3). The names and Accession Numbers of previously reported Japanese PPV-D isolates used in this study are as follows: Ad1, LC374954; Ad2, LC374955; Ad3, LC374956; Ak1, AB576045; Ak2, AB576046; Ak3, AB576047; Ak4, LC374957; Ak5, LC374958; Ak6, LC374959; Ak7, LC374960; Ak8, LC374961; Ak9, LC374962; As1, LC374963; As2, LC374964; As3, LC374965; Fu1, LC374968; Ha1, AB576048; Ha2, AB576049; Ha3, AB576050; Ha4, AB576051; Ha5, LC374969; Ha6, LC374970; Ha7, LC374971; Hi1, AB576052; Hi2, AB576053; Hi3, AB576054; Hi4, AB576055; Hk1, LC374973; Hk2, LC374977; Hk6, LC374978; Hk7, LC374979; Hk8, LC374980; Hk10, LC374974; Hk12, LC374975; Hk13, LC374976; Ko2, LC375026; Ok1, AB576056; Ok2, AB576057; Ok3, LC375051; Ou1, AB545926; Ou2, AB576058; Ou3, AB576059; Ou4, AB576060; Ou5, AB576061; Ou6, AB576062; Ou7, AB576063; Ou8, AB576064; Ou9, AB576065; Ou12, AB576066; Ou13, AB576067; Ou14, AB576068; Ou15, AB576069; Ou16, AB576070; Ou17, AB576071; Ou18, AB576072; Ou19, LC375056; Ou20, LC375057; Ou25, LC375058; Ou26, LC375059; Ou27, LC375060; Ou28, LC375061; Ou29, LC375062; Ou31, LC375063; Ou32, LC375064; Ou36, LC375065; Ou38, LC375066; Ou39, LC375067; Ou40, LC375068; Ou41, LC375069; OuX1-3, LC375070; OuX2-6, LC375080; OuX3-6, LC375082; OuX4-1, LC375083; OuX4-4, LC375084; OuX5-1, LC375086; OuX5-5, LC375087; OuX6-5, LC375089; OuX8-1, LC375090; OuX8-8, LC375091; OuX10-1, LC375071; OuX10-3, LC375072; OuX10-4, LC375073; OuX11-10, LC375074; OuX11-12, LC375075; OuX11-14, LC375076; OuX12-1, LC375077; OuX14-12, LC375078; OuX15-4, LC375079; OuX20-1, LC375081; OuX42-5, LC375085; OuX53-8, LC375088; OuXN, LC375092; Kg1, LC375018; Kg2, LC375019; Kg3, LC375020; Kg4, LC375021; Kg5, LC375022; Kg6, LC375023; Kg7, LC375024; Mi1, AB576073; Mi2, AB576074; Mi3, AB576075; Mi4, LC375033; Mi5, LC375034; Mi6, LC375035; Mi10, LC375030; Mi12, LC375031; Mi13, LC375032; Od1, AB576076; Od2, AB576077; Od3, AB576078; Od4, AB576079; Od5, AB576080; Hn1, LC374981; Hn2, LC374982; Ir1, LC374991; Ni1, LC375049; Ni2, LC375050; Fs2, LC374967; In1, LC374984; In2, LC374985; In3, LC374986; In4, LC374987; In5, LC374988; In6, LC374989; In9, LC374990; Ts1, LC375116; Ts2, LC375117; Ts3, LC375118; Ts4, LC375119; Ng1, LC375041; Ng2, LC375042; Ng3, LC375043; Ng4, LC375044; Ng5, LC375045; Ng6, LC375046; Ng7, LC375047; Ng8, LC375048; Na1, LC375036; Na2, LC375037; Na3, LC375038; Na4, LC375039; Na5, LC375040; Sa1, LC375093; Sa2, LC375094; Sa4, LC375096; Sa5, LC375097; Sa22, LC375095; Sa57, LC375098; Ch1, LC374966; Hg1, LC374972; Iz1, LC375015; Iz2, LC375016; Kc1, LC375017; Kn3, LC375025; Ks1, LC375027; Os1, LC375052; Os2, LC375053; Os3, LC375054; Os5, LC375055; Sk3, LC375100; Su1, LC375101; Su3, LC375103; Su14, LC375102; Su46, LC375104; Su49, LC375105; Su81, LC375106; Su83, LC375107; Tn1, LC375110; Tn10, LC375111; Tn29, LC375112; Tn43, LC375113; Tn62, LC375114; Tn84, LC375115; Ty1, LC375120; Ya1, LC375126; Wa2, LC375121; Wa3, LC375122; Wa4, LC375123; Wa6, LC375124; Wa7, LC375125; Ig3339, LC374983; It2, LC374998; It5, LC375010; It6, LC375012; It146, LC374994; It386, LC375007; It588, LC375011; It712, LC375013; It953, LC375014; It1366, LC374992; It1422, LC374993; It1956, LC374995; It1961, LC374996; It1982, LC374997; It2079, LC374999; It2166, LC375000; It2188, LC375001; It2433, LC375002; It2508, LC375003; It2763, LC375004; It3114, LC375005; It3171, LC375006; It3929, LC375008; It4141, LC375009; Kw2694, LC375028; Kw4813, LC375029; Sd3768, LC375099; Tk5466, LC375108; Tk5502, LC375109 (see the Additional file 9: Table S5). All data generated or analyzed during the current study are included in this published article and its Additional files. Voucher specimens cannot be deposited because the movements of any PPV-infected samples including infected leaves are prohibited under the MAFF’s ordinance (www.pps.go.jp/english/pestreport/index.html).

## Abbreviation

PPV: *Plum pox virus*
