# Supplementary material for: Genome sequence analysis of new plum pox virus isolates from Japan
Source: BMC Res Notes. 2021 Jul 10;14:266. doi: 10.1186/s13104-021-05683-9 (PMC8272314; doi:10.1186/s13104-021-05683-9)
Supplement: Supplementary file 4 — Additional file 4: Figure S1. Map of the collection locations of new isolates and previously reported Japanese PPV-D isolates used for analysis or discussion in this study. [file 13104_2021_5683_MOESM4_ESM.pdf]

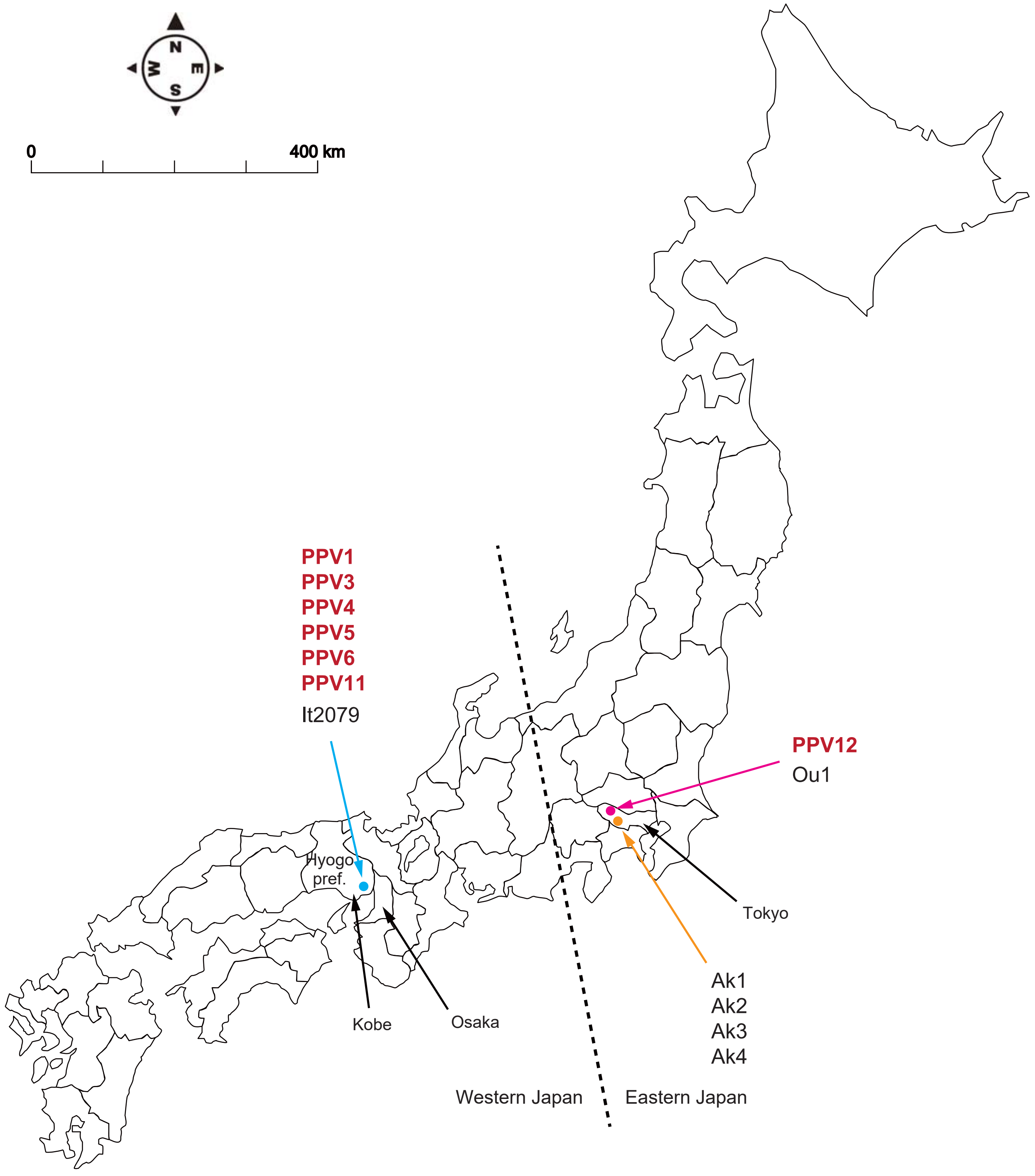

Fig. S1 Map of the collection locations (shown as colored dots) of new isolates (shown as red letters) and previously reported Japanese PPV-D isolates used for analysis or discussion in this study
