# Supplementary material for: Genome sequence analysis of new plum pox virus isolates from Japan
Source: BMC Res Notes. 2021 Jul 10;14:266. doi: 10.1186/s13104-021-05683-9 (PMC8272314; doi:10.1186/s13104-021-05683-9)
Supplement: Supplementary file 6 — Additional file 6: Figure S2. Phylogenetic tree generated by the maximum-likelihood method with 1000 bootstrap replicates based on complete genome sequences of new seven isolates and each representative isolate of PPV strains. [file 13104_2021_5683_MOESM6_ESM.pdf]

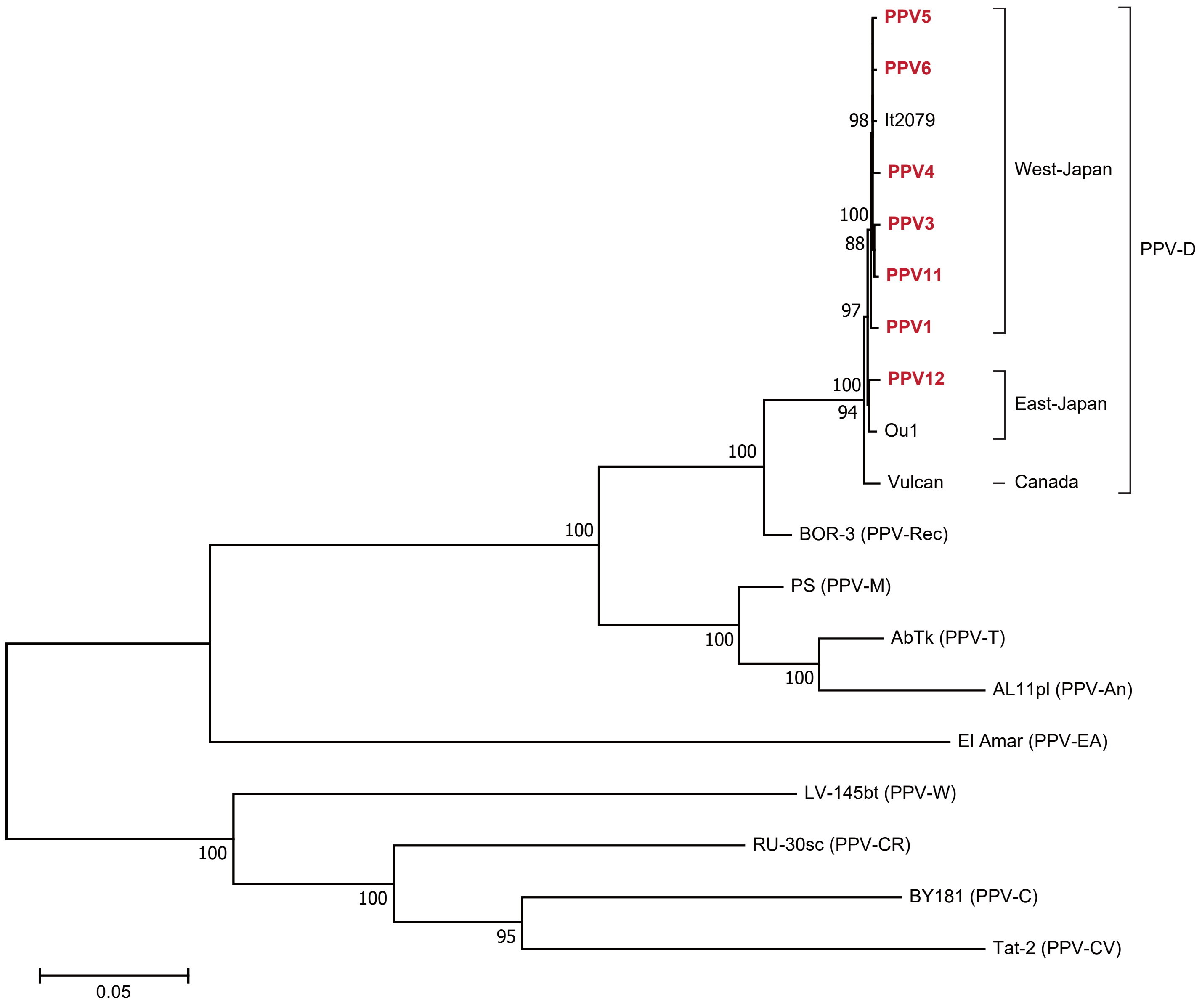

Fig. S2 Phylogenetic tree generated by the maximum-likelihood method with 1000 bootstrap replicates based on complete genome sequences of new seven isolates (shown as red letters) and each representative isolate of PPV strains. Branch lengths indicate the number of nucleotide differences per site, and numbers at nodes indicate bootstrap values
