# Supplementary material for: Genome sequence analysis of new plum pox virus isolates from Japan
Source: BMC Res Notes. 2021 Jul 10;14:266. doi: 10.1186/s13104-021-05683-9 (PMC8272314; doi:10.1186/s13104-021-05683-9)

Fig. S3 Phylogenetic tree generated by the maximum-likelihood method with 1000 bootstrap replicates based on complete genome sequences of new seven isolates (shown as red letters) and 47 non-Japanese PPV-D isolates. Two PPV-M isolates (PS: AJ243957 and SK 68: M92280) were used as an outgroup. Branch lengths indicate the number of nucleotide differences per site, and numbers at nodes indicate bootstrap values greater than 70

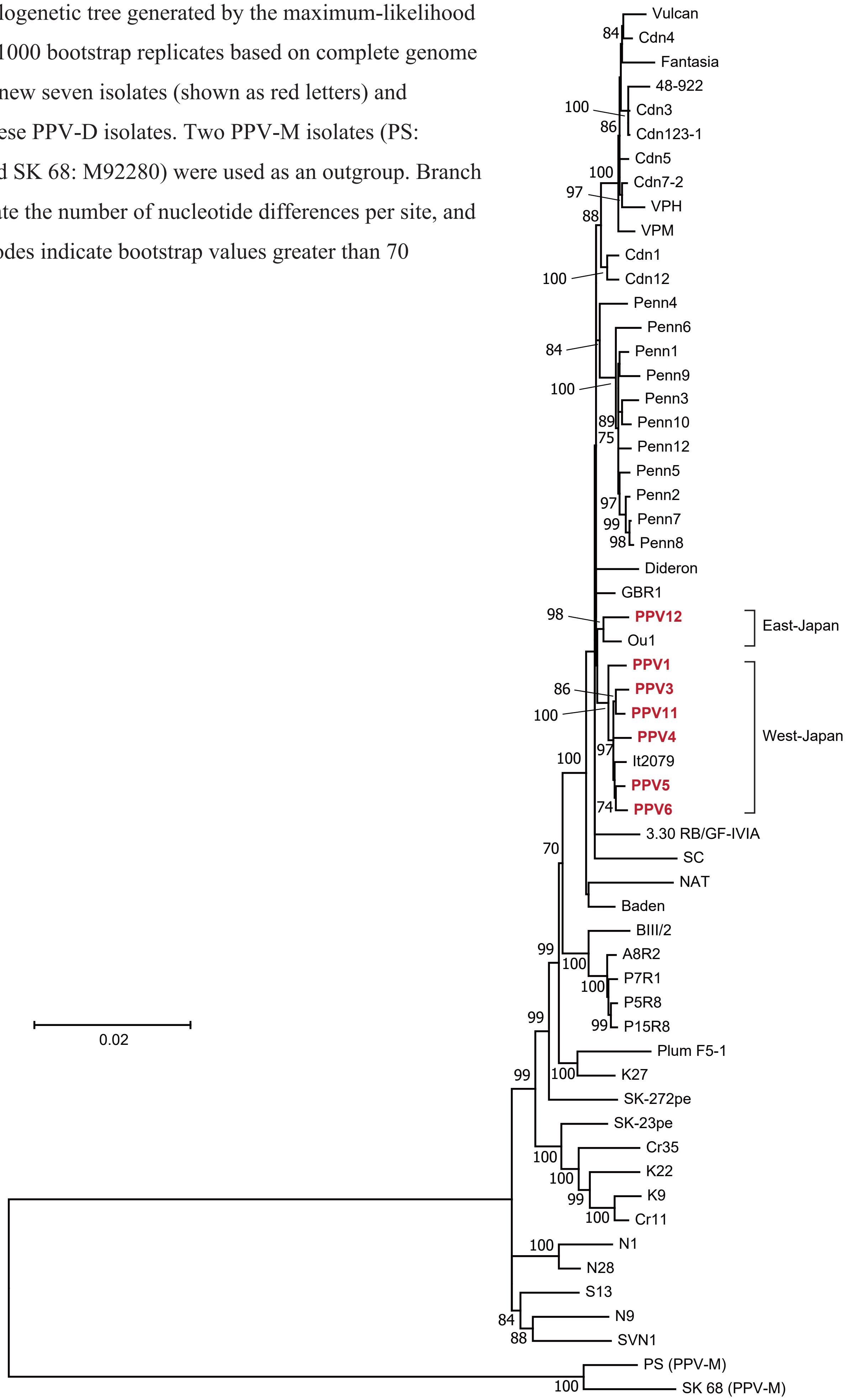

Supplement: Supplementary file 7 — Additional file 7: Figure S3. Phylogenetic tree generated by the maximum-likelihood method with 1000 bootstrap replicates based on complete genome sequences of new seven isolates and 47 non-Japanese PPV-D isolates. [file 13104_2021_5683_MOESM7_ESM.pdf]
