# Supplementary material for: Genome sequence analysis of new plum pox virus isolates from Japan
Source: BMC Res Notes. 2021 Jul 10;14:266. doi: 10.1186/s13104-021-05683-9 (PMC8272314; doi:10.1186/s13104-021-05683-9)
Supplement: Supplementary file 8 — Additional file 8: Figure S4. Multiple amino acid sequence alignment of Japanese PPV-D isolates and 47 non-Japanese PPV-D isolates around the 1407th amino acid residues. [file 13104_2021_5683_MOESM8_ESM.docx]

aa1407

Japanese isolates

**PPV1** DFKTQKEVKVIVEESLSFQQFVSNLGTGCNSDILKHGVNVLVYVASYNEVDTLSKLLTDR 1439

**PPV3** DFKTQKEVKVIVEESLSFQQFVSNLGTSCNSDILKHGVNVLVYVASYNEVDTLSKLLTDR 1439

**PPV4** DFKTQKEVKVIVEESLSFQQFVSNLGTSCNSDILKHGVNVLVYVASYNEVDTLSKLLTDR 1439

**PPV5** DFKTQKEVKVIVEESLSFQQFVSNLGTSCNSDILKHGVNVLVYVASYNEVDTLSKLLTDR 1439

**PPV6** DFKTQKEVKVIVEESLSFQQFVSNLGTSCNSDILKHGVNVLVYVASYNEVDTLSKLLTDR 1439

**PPV11** DFKTQKEVKVIVEESLSFQQFVSNLGTSCNSDILKHGVNVLVYVASYNEVDTLSKLLTDR 1439

**PPV12** DFKTQKEVKVIVEESLSFQQFVSNLGTGCNSDILKHGVNVLVYVASYNEVDTLSKLLTDR 1439

Ou1 DFKTQKEVKVIVEESLSFQQFVSNLGTGCNSDILKHGVNVLVYVASYNEVDTLSKLLTDR 1439

It2079 DFKTQKEVKVIVEESLSFQQFVSNLGTSCNSDILKHGVNVLVYVASYNEVDTLSKLLTDR 1439

Non-Japanese isolates

N1 DFKTQKEVKVIVEESLSFQQFVSNLGTGCNSDILKHGVNVLVYVASYNEVDTLSKLLTDR 1439

N9 DFKTQKEVKVIVEESLSFQQFVSNLGTGCNSDILKHGVNVLVYVASYNEVDTLGKLLTDR 1439

N28 DFKTQKEVKVIVEESLSFQQFVSNLGTGCNSDILKHGVNVLVYVASYNEVDTLSKLLTDR 1439

Fantasia DFKTQKEVKVIVEESLSFQQFVSNLGTGCNSDILKHGVNVLVYVASYNEVDTLSKLLTDR 1439

Vulcan DFKTQKEVKVIVEESLSFQQFVSNLGTGCNSDILKHGVNVLVYVASYNEVDTLSKLLTDR 1439

48-922 DFKTQKEVKVIVEESLSFQQFVSNLGTGCNSDILKHGVNVLVYVASYNEVDTLSKLLTDR 1439

Cdn1 DFKTQKEVKVIVEESLSFQQFVSNLGTGCNSDILKHGVNVLVYVASYNEVDTLSKLLTDR 1439

Cdn3 DFKTQKEVKVIVEESLSFQQFVSNLGTGCNSDILKHGVNVLVYVASYNEVDTLSKLLTDR 1439

Cdn4 DFKTQKEVKVIVEESLSFQQFVSNLGTGCNSDILKHGVNVLVYVASYNEVDTLSKLLTDR 1439

Cdn5 DFKTQKEVKVIVEESLSFQQFVSNLGTGCNSDILKHGVNVLVYVASYNEVDTLSKLLTDR 1439

Cdn7-2 DFKTQKEVKVIVEESLSFQQFVSNLGTGCNSDILKHGVNVLVYVASYNEVDTLSKLLTDR 1439

Cdn12 DFKTQKEVKVIVEESLSFQQFVSNLGTGCNSDILKHGVNVLVYVASYNEVDTLSKLLTDR 1439

Cdn123-1 DFKTQKEVKVIVEESLSFQQFVSNLGTGCNSDILKHGVNVLVYVASYNEVDTLSKLLTDR 1439

VPH DFKTQKEVKVIVEESLSFQQFVSNLGTGCNSDILKHGVNVLVYVASYNEVDTLSKLLTDR 1439

VPM DFKTQKEVKVIVEESLSFQQFVSNLGTGCNSDILKHGVNVLVYVASYNEVDTLSKLLTDR 1439

Dideron DFKTQKEVKVIVEESLSFQQFVSNLGTGCNSDILKHGVNVLVYVASYNEVDTLSKLLTDR 1440

NAT EFKTQKEVKVIVEESLSFQQFVSNLGTGCNSDILKHGVNVLVYVASYNEVDTLSKLLTDR 1439

Baden DFKTQKEVKVIVEESLSFQQFVSNLGTGCNSDILKHGVNVLVYVASYNEVDTLSKLLTDR 1439

P5R8 DFKTQKEVKVIVEESLSFQQFVSNLGTGCNSDILKHGVNVLVYVASYNEVDTLSKLLTDR 1439

P7R1 DFKTQKEVKVIVEESLSFQQFVSNLGTGCNSDILKHGVNVLVYVASYNEVDTLSKLLTDR 1439

A8R2 DFKTQKEVKVIVEESLSFQQFVSNLGTGCNSDILKHGVNVLVYVASYNEVDTLSKLLTDR 1439

P15R8 DFKTQKEVKVIVEESLSFQQFVSNLGTGCNSDILKHGVNVLVYVASYNEVDTLSKLLTDR 1439

SC DFKTQKEVKVIVEESLSFQQFVSNLGTGCNSDILKHGVNVLIYVASYNEVDTLSKLLTDR 1439

S13 DFKTQKEVKVIVEESLSFQQFVSNLGTGCNSDILKHGVNVLVYVASYNEVDTLSKLLTDR 1439

Blll/2 DFKTQKEVKVIVEESLSFQQFVSNLGTGCNSDILKHGVNVLVYVASYNEVDTLSKLLTDR 1439

SK-272pe DFKTQKEVKVIVEESLSFQQFVSNLGTGCNSDILKHGVNVLVYVASYNEVDTLSKLLTDR 1439

SK-23pe DFKTQKEVKVIVEESLSFQQFVSNLGTGCNSDILKHGVNVLVYVASYNEVDTLSKLLTDR 1439

SVN1 DFKTQKEVKVIIEESLSFQQFVSNLGTGCNSDILKHGVNVLVYVASYNEVDTLGKLLTDR 1439

Plum_F5-1 DFKTQKEVKVIVEESLSFQQFVSNLGTGCNSDILKHGVNVLVYVASYNEVDTLSKLLTDR 1439

3.30_RB/GF-IVIA DFKTQKEVKVIVEESLSFQQFVSNLGTGCNSDILKHGVNVLVYVASYNEVDTLSKLLTDR 1439

K9 DFKTQKEVKVIVEESLSFQQFVSNLGTGCNSDILKHGVNVLVYVASYNEVDTLSKLLTDR 1439

K22 DFKTQKEVKVIVEESLSFQQFVSNLGTGCNSDILKHGVNVLVYVASYNEVDTLSKLLTDR 1439

Cr11 DFKTQKEVKVIVEESLSFQQFVSNLGTGCNSDILKHGVNVLVYVASYNEVDTLSKLLTDR 1439

Cr35 DFKTQKEVKVIVEESLSFQQFVSNLGTGCNSDILKHGVNVLVYVASYNEVDTLSKLLTDK 1439

K27 DFKTQKEVKVIVEESLSFQQFVSNLGTGCNSDILKHGVNVLVYVASYNEVDTLSKLLTDR 1439

GBR1 DFKTQKEVKVIVEESLSFQQFVSNLGTGCNSDILKHGVNVLVYVASYNEVDTLSKLLTDR 1439

Penn1 DFKTQKEVKVIVEESLSFQQFVSNLGTGCNSDILKHGVNVLVYVASYNEVDTLSKLLTDR 1439

Penn2 DFKTQKEVKVIVEESLSFQQFVSNLGTGCNSDILKHGVNVLVYVASYNEVDTLSKLLTDR 1439

Penn3 DFKTQKEVKVIVEESLSFQQFVSNLGTGCNSDILKHGVNVLVYVASYNEVDTLSKLLTDR 1439

Penn4 DFKTQKEVKVIVEESLSFQQFVSNLGTGCNSDILKHGVNVLVYVASYNEVDTLSKLLTDR 1439

Penn5 DFKTQKEVKVIVEESLSFQQFVSNLGTGCNSDILKHGVNVLVYVASYNEVDTLSKLLTDR 1439

Penn6 DFKTQKEVKVIVEESLSFQQFVSNLGTGCNSDILKHGVNVLIYVASYNEVDTLSKLLTDR 1439

Penn7 DFKTQKEVKVIVEESLSFQQFVSNLGTGCNSDILKHGVNVLVYVASYNEVDTLSKLLTDR 1439

Penn8 DFKTQKEVKVIVEESLSFQQFVSNLGTGCNSDILKHGVNVLVYVASYNEVDTLSKLLTDR 1439

Penn9 DFKTQKEVKVIVEESLSFQQFVSNLGTGCNSDILKHGVNVLVYVASYNEVDTLSKLLTDR 1439

Penn10 DFKTQKEVKVIVEESLSFQQFVSNLGTGCNSDILKHGVNVLVYVASYNEVDTLSKLLTDR 1439

Penn12 DFKTQKEVKVIVEESLSFQQFVSNLGTGCNSDILKHGVNVLVYVASYNEVDTLSKLLTDR 1439

:**********:***************.*************:***********.*****:

**Fig. S4** Multiple amino acid sequence alignment of Japanese PPV-D isolates and 47 non-Japanese PPV-D isolates around the 1407th amino acid residues. The amino acid residues at 1407 are highlighted with light blue. Symbols denote the degree of conservation observed in each column: “*” (identical residues in all sequence), “:” (highly conserved column), “.” (weakley conserved column)
